# Measurement reactivity in ambulatory assessment: Increase in emotional clarity over time independent of sampling frequency

**DOI:** 10.3758/s13428-024-02346-y

**Published:** 2024-01-30

**Authors:** Charlotte Ottenstein, Kilian Hasselhorn, Tanja Lischetzke

**Affiliations:** RPTU Kaiserslautern-Landau, Fortstr. 7, 76829 Landau, Germany

**Keywords:** Measurement reactivity, Ambulatory assessment, Emotional clarity, Mood, Mood regulation

## Abstract

Ambulatory assessment (AA) studies are frequently used to study emotions, cognitions, and behavior in daily life. But does the measurement itself produce reactivity, that is, are the constructs that are measured influenced by participation? We investigated individual differences in intraindividual change in momentary emotional clarity and momentary pleasant-unpleasant mood over the course of an AA study. Specifically, we experimentally manipulated sampling frequency and hypothesized that the intraindividual change over time would be stronger when sampling frequency was high (vs. low). Moreover, we assumed that individual differences in dispositional mood regulation would moderate the direction of intraindividual change in momentary pleasant-unpleasant mood over time. Students (*n* = 313) were prompted either three or nine times a day for 1 week (data collection took place in 2019 and 2020). Multilevel growth curve models showed that momentary emotional clarity increased within participants over the course of the AA phase, but this increase did not differ between the two sampling frequency groups. Pleasant-unpleasant mood did not show a systematic trend over the course of the study, and mood regulation did not predict individual differences in mood change over time. Again, results were not moderated by the sampling frequency group. We discuss limitations of our study (e.g., WEIRD sample) and potential practical implications regarding sampling frequency in AA studies. Future studies should further systematically investigate the circumstances under which measurement reactivity is more likely to occur.

In the fields of psychology and life science, the use of ambulatory assessment (AA) is increasing (Hamaker & Wichers, 2017). AA, also referred to as daily diary, experience sampling, or ecological momentary assessment, is a method for assessing individuals' daily life experiences, encompassing their ongoing behavior, experience, physiology, and environmental aspects in naturalistic and unconstrained settings (Fahrenberg et al., 2007; Mehl & Conner, 2012). Whereas there are many clear advantages of AA – such as high ecological validity (e.g., Trull & Ebner-Priemer, 2013), the possibility to study within-person dynamics (e.g., Hamaker & Wichers, 2017), or reduced recall bias (for an overview, see Conner & Feldman Barrett, 2012) – it is still unclear whether (or under which conditions) the repeated assessments affect the constructs that are measured.

## Measurement reactivity in ambulatory assessment studies

Measurement reactivity pertains to the question of whether psychological measurement influences the (self-reports of the) constructs that are measured (French & Sutton, 2010; Shiffman et al., 2008). It can also occur in one-time assessments (Webb et al., 2000), but it is especially relevant in AA studies in which participants answer the same questions repeatedly. When answering an AA questionnaire, participants read the items, think about their meaning, monitor their behavior, cognitions, or emotions, and finally choose a response option (Tourangeau et al., 2000). Whereas the attention a person pays to the constructs that are being measured is naturally enhanced during the AA prompts, the participant’s behavior, cognitions, or emotions might also be altered during the whole study period (or even after the study period) due to heightened self-monitoring (Barta et al., 2012). It is typical in AA studies to present the same questions repeatedly. After answering the first prompts, participants typically know which items will be presented next and might change their behavior, cognitions, or emotions in expectation of the next prompt. Measurement reactivity should be stronger when the daily sampling frequency is high because participants are confronted with the items more often, a phenomenon that should in turn enhance self-monitoring.

Whereas AA studies can be seen as interventions that might enhance favorable characteristics, it can be problematic when unfavorable characteristics are studied in clinical or other unstable samples (e.g., suicidality among teens; Czyz et al., 2018). Moreover, the validity of the data might be compromised if measurement reactivity occurs (Barta et al., 2012; Buu et al., 2020). Hence, it is important to study measurement reactivity in the context of AA studies. Measurement reactivity might depend on the constructs being studied, participant characteristics, or study design. Our aim was to investigate measurement reactivity regarding momentary emotional clarity (the extent to which individuals can unambiguously identify and label their affective experiences) and momentary pleasant-unpleasant mood. For this purpose, we experimentally manipulated the number of daily prompts (i.e., sampling frequency) to investigate whether measurement reactivity increased when sampling frequency was high.

Measurement reactivity is usually investigated in terms of mean-level change in the measured constructs over the course of an AA study, but empirical findings on mean-level change have been mixed. Studies have shown measurement reactivity with respect to increased suicidality among teens (Czyz et al., 2018), increased alcohol use (Buu et al., 2020), and increased actual prescription drug misuse (Papp et al., 2020). Measurement reactivity has sometimes been found to occur in only some subgroups. For example, measurement reactivity regarding increased parent–child conflicts and warmth occurred only in parent reports but not in child reports (Reynolds et al., 2016). However, most studies have revealed no measurement reactivity with respect to pain (Kratz et al., 2017; Stone et al., 2003), body dissatisfaction (Heron & Smyth, 2013), attitudes (Heron & Smyth, 2013), stress (Pryss et al., 2019), alcohol consumption (Hufford et al., 2002; Labhart et al., 2020), or rumination (Eisele et al., 2023).

Empirical findings on measurement reactivity with respect to affect and the precise representation of affect (for an overview of the constructs included under this label, see Kashdan et al., 2015) have also been inconclusive. One study showed decreased positive affect (Eisele et al., 2023). Moreover, positive affect (happy mood) decreased in groups with recent or past suicidal attempts but not in affective or healthy control groups (Husky et al., 2014). However, most studies have found no measurement reactivity regarding positive affect (Aaron et al., 2005; Cruise et al., 1996; De Vuyst et al., 2019; Helbig et al., 2009; Husky et al., 2010) or negative affect (Aaron et al., 2005; Cruise et al., 1996; De Vuyst et al., 2019; Eisele et al., 2023; Helbig et al., 2009; Heron & Smyth, 2013; Husky et al., 2010). Regarding the precise representation of affect, two studies showed an increase in emotion differentiation (Hoemann et al., 2021; Widdershoven et al., 2019), one study showed an increase in emotional awareness (Kauer et al., 2012), and yet another study showed a decrease in emotional awareness but no measurement reactivity for emotional clarity (Eisele et al., 2023).

## Predicting measurement reactivity

The diverse empirical findings suggest that whether measurement reactivity occurs during an AA study seems to depend on the construct and on sample characteristics. Moreover, characteristics of the study design might also play a crucial role. It can be assumed that more blatantly confronting participants with items pertaining to a certain construct of interest enhances measurement reactivity (e.g., due to heightened self-monitoring). The amount of confrontation can be heightened by increasing study length (i.e., more days with AA), sampling frequency (i.e., more measurement occasions per day), or questionnaire length (i.e., more items per AA questionnaire). Only a few studies have experimentally manipulated questionnaire length (Eisele et al., 2023) or sampling frequency (Conner & Reid, 2012; Eisele et al., 2023; McCarthy et al., 2015; Stone et al., 2003). On the one hand, Eisele et al. (2023) found no effect of questionnaire length or sampling frequency on change in emotional awareness, emotional clarity, rumination, or positive or negative affect. Other studies also found no effect of sampling frequency on change in pain (Stone et al., 2003), the desire to smoke, anxiety, anger, hunger, or positive affect (McCarthy et al., 2015). On the other hand, Conner and Reid (2012) showed that change in happiness depended on sampling frequency and individual characteristics. Participants low in depression or low in neuroticism showed an increase in happiness when sampling frequency was high. In turn, participants high in depression or high in neuroticism showed an increase in happiness when sampling frequency was low.

## The current study

The diverse empirical results highlight the need to study measurement reactivity for each construct separately and to incorporate individual and study characteristics. Our aim was to investigate whether momentary emotional clarity and momentary pleasant-unpleasant mood change over the course of an AA study (as an indication of measurement reactivity) and contribute to an understanding of the circumstances (design characteristics of the AA study, individual characteristics) in which measurement reactivity effects might be more likely to arise. We experimentally manipulated sampling frequency and included mood regulation competence as an individual characteristic.

### Measurement reactivity regarding momentary emotional clarity

Emotional clarity represents the extent to which individuals can unambiguously identify and label their affective experiences (e.g., Gohm & Clore, 2002; Salovey et al., 1995). Being asked repeatedly to indicate one’s level of different mood dimensions could help individuals discriminate between different affective states (Widdershoven et al., 2019), and potentially lead individuals to be clearer (more certain) about what they feel. Moreover, by repeatedly answering questions about the momentary situational context (e.g., presence of other people, current place, stress level) as well as the affect they are experiencing in an AA study, participants might reflect more on the sources of their affect (Boden & Berenbaum, 2011), and such reflection might help them gain more emotional clarity over time. A higher number of these kinds of practice opportunities should strengthen the increase in emotional clarity. Thus, we hypothesized that the group with a higher sampling frequency (i.e., the group with more measurement occasions in which they are asked to report on their current mood) should show a larger increase in momentary emotional clarity over time than the group with a lower sampling frequency.

### Measurement reactivity regarding momentary pleasant-unpleasant mood

Participating in an AA study that includes questions about affect goes along with heightened attention to feelings. This increased attention to feelings might have an impact on affect itself. For individuals low in affect regulation (i.e., a low ability to repair negative affective states and a low ability to actively maintain positive affective states), paying more attention to negative feelings could induce rumination and promote mood-congruent information processing, hence worsening affect over time. But individuals high in affect regulation could make use of an increase in their attention to feelings by effectively improving a bad mood or downregulating a negative emotion at an early stage or, in the case of a positive affective state, by engaging in active strategies to maintain this positive state before it fades. In line with this theorizing, Lischetzke and Eid (2003) found that dispositional attention to feelings was positively related to dispositional pleasant-unpleasant mood for individuals with high mood regulation competence, whereas attention to feelings was negatively related to dispositional pleasant-unpleasant mood for individuals with low mood regulation competence. Accordingly, with regard to the heightened attention to momentary feelings that an AA study induces, its effect on affect itself should depend on participants’ affect regulation competence: The mood of participants with low mood regulation competence should worsen across the time of the study, and the mood of participants with high mood regulation competence should improve. As the frequency with which individuals pay attention to their feelings should be higher in the group with the high sampling frequency, the moderating effect of mood regulation competence in this group should be stronger than in the group with the low sampling frequency.

#### *Hypotheses*

First, the group with the higher sampling frequency was expected to show a larger increase in momentary emotional clarity over time than the group with the lower sampling frequency (Hypothesis 1). Second, individuals with lower mood regulation competence were expected to show a decrease in momentary pleasant-unpleasant mood over time, and individuals with higher mood regulation competence were expected show an increase in momentary pleasant-unpleasant mood over time (Hypothesis 2a). This difference between individuals with low versus high mood regulation competence was expected to be moderated by the experimental group: The increase/decrease in momentary pleasant-unpleasant mood over time was expected be more pronounced in the high sampling frequency group than in the low sampling frequency group (Hypothesis 2b).

## Method

### Study design

The whole study consisted of an initial online survey, 2 weeks of AA, and two retrospective online surveys that followed immediately after each of the 2 AA weeks. During the first AA week, participants were prompted either three (low sampling frequency group) or nine (high sampling frequency group) times a day (random assignment to one experimental condition). Participants chose one out of two time schedules that best fit their waking hours (9:00–21:00 or 10:30–22:30). The nine prompts in the high sampling frequency group were distributed evenly across the day. The three prompts in the low sampling frequency group were scheduled at the same time of day as the first, fifth, and ninth prompts of the high sampling frequency group. During the second AA week, the sampling frequency was switched between the groups. The reason for the switch was to guarantee that all participants spent an equivalent amount of time taking part in the study, making the financial compensation fair for both groups. Because the main emphasis was on the between-group comparison (high vs. low sampling frequency), rather than analyzing the effects of switching sampling frequencies within persons, the analyses presented in this paper were based on data obtained during the initial AA week (and the initial online survey). The initial online survey assessed demographic information and trait self-report measures (all items can be found in the codebook on the OSF). During the AA phase, measures of momentary motivation, time pressure, momentary mood, momentary emotional clarity, state personality, stress, perceived burden, and items for assessing the characteristics of the present situation (current place, presence of other individuals) were included. The additional six occasions per day in the high sampling frequency group contained only items that pertained to the present situation, mood, and state personality.

### Participants and procedure

All study procedures were approved by the psychological ethics committee at the University of Koblenz-Landau, Germany (now RPTU Kaiserslautern-Landau, Germany). Participants were required to be students and to own an Android smartphone. They were recruited via flyers, posters, e-mails, and posts on Facebook during the students’ semester breaks in spring 2019 and spring 2020 (during the non-lecture period between the winter semester and the summer semester). After informed consent was obtained, the study began with the initial online survey. Afterwards, participants were randomly assigned to one of two experimental conditions (low sampling frequency vs. high sampling frequency) and randomly assigned to a starting day of the week. The administration of the AA phase was done via the smartphone application movisensXS (Versions 1.4.5, 1.4.6, 1.4.8, 1.5.0, and 1.5.1; movisens GmbH, Karlsruhe, Germany). Participants were given 15€ if they answered at least 50% of the AA questionnaires and were given the chance to win 25€ extra if they answered at least 80% of the AA questionnaires. Additionally, they could receive personal feedback on the constructs measured in the study after their participation was complete.

The current research was part of a larger study (Hasselhorn et al., 2022). As most hypotheses in this study focused on group differences, we based our sample size considerations on the power to detect a small-to-moderate (*d* = 0.30) mean difference (independent-samples *t* test, one-tailed). We needed 278 participants to achieve a power of .80. A total of 474 individuals filled out the initial online survey. Due to technical problems with the smartphone application for the AA phase, various participants withdrew their participation before the AA phase. A total of 318 individuals took part in the first AA week that followed. Data from five participants (three in the high sampling frequency group) were excluded from the analyses because they indicated that their data should not be used in the analyses. Subsequently, we removed 330 AA questionnaires (149 AA questionnaires in the low sampling frequency group) due to inconsistent responding (Meade & Craig, 2012) across the reverse-poled (mood) items.^1^ Therefore, the final sample consisted of 313 students (low sampling frequency group: *n* = 153; 86% women; age range: 18 to 34 years, *M* = 23.18, *SD* = 3.23; high sampling frequency group: *n* = 160; 83% women; age range: 18 to 40 years, *M* = 23.98, *SD* = 4.12), providing 8778 AA questionnaires (low sampling frequency group: 1 to 23 AA questionnaires, *M* = 14.00, *SD* = 5.33; high sampling frequency group: 2 to 63 AA questionnaires, *M* = 41.48, *SD* = 15.59).^2^^,^ 3 A sensitivity analysis revealed that we were able to detect an effect size of *d* = 0.28 with a power of .80 (one-tailed test) with the final sample of 313 students.

### Measures

#### Sampling frequency

A dichotomous factor was used to indicate the sampling frequency (0 = *low sampling frequency group*, 1 = *high sampling frequency group*).

#### Momentary pleasant-unpleasant mood

We measured momentary pleasant-unpleasant mood with an adapted short version of the Multidimensional Mood Questionnaire (Steyer et al., 1997) that has been used in previous AA studies (e.g., Lischetzke et al., 2012; Lischetzke et al., 2022). Participants indicated how they felt at that moment on four items (bad-good [reverse-scored], unwell-well, unhappy-happy [reverse-scored], and unsatisfied-satisfied). The response format was a seven-point Likert scale with each pole labeled (e.g., 1 = *very unwell* to 7 = *very well*). We calculated a mean score across the items so that a higher score indicated more pleasant-unpleasant mood. The within-person ω (Geldhof et al., 2014) was .91, and the between-person ω was .99.

#### Momentary emotional clarity

Directly after answering the momentary mood items, participants rated their amount of momentary emotional clarity three times a day. In the high sampling frequency group, this was done on the first, fifth, and ninth measurement occasions of the day to ensure that the two experimental groups answered the emotional clarity items with a similar frequency. As a measure of momentary emotional clarity, we assessed participants’ confidence in their momentary mood ratings, which has been shown to converge with an indirect, response-time-based measure of emotional clarity and to be positively correlated with dispositional emotional clarity on the person level (Lischetzke et al., 2005, 2011). We used two items that were answered on seven-point Likert scales with each pole labeled (‘How easy or difficult was it for you to rate your momentary mood?’, 1 = *very difficult* to 7 = *very easy*; ‘How certain or uncertain were you when rating your momentary mood?’, 1 = *very certain* to 7 = *very uncertain* [reverse-scored]). The two items were averaged to form a scale score. In the present study, aggregated momentary emotional clarity was correlated with dispositional emotional clarity (as measured with a validated German scale by Lischetzke et al., 2001), *r* = .40, *p* < .001. We estimated local (within-occasion) reliability (Buse & Pawlik, 1996) because the momentary emotional clarity measure consisted of only two items. This was done by calculating the polychoric correlation between the items for each measurement occasion and summarizing them by identifying the median. The median polychoric correlation across measurement occasions was .70. Local reliability indicates the internal consistency of the measure at the same occasion, whereas aggregate reliability indicates the consistency of aggregate scores across occasions. To estimate aggregate reliability, we calculated the Pearson correlation between the two items (aggregated across occasions), which was .68.

#### Dispositional mood regulation

Two dimensions of mood regulation effectiveness were assessed with the mood regulation scale by Lischetzke and Eid (2003) during the initial online survey. The *negative mood repair* (NMR) subscale included six items (e.g., ‘It is easy for me to improve my bad mood’), and the *positive mood maintenance* subscale (PMM) included five items (e.g., ‘When I am in a good mood, I am able to stay that way for a long time’). All items were answered on 4-point response scales (1 = *strongly disagree* to 4 = *strongly agree*). McDonald’s ω (McDonald, 1999; computed via the omega function from the psych package, Revelle, 2023; also referred to as Revelle’s ω total, McNeish, 2018) was .84 for NMR and .81 for PMM.

### Data analytic methods

To test our hypotheses, we used multilevel growth curve models with measurement occasions (at Level 1) nested in persons (at Level 2). Day of the study was used as the time variable (0 = first day of the study). We did not use running numbering for all measurement occasions (1–63) to circumvent biases due to diurnal mood patterns (Stone et al., 1996). Random slopes were specified for this predictor (i.e., participants were allowed to differ in intraindividual change over time). To analyze whether momentary emotional clarity changed within participants over the course of the study, we began with an unconditional growth curve model (with the day of the study as the only predictor; Model 0). To test Hypothesis 1 (effect of sampling frequency on change in clarity over time), we entered the sampling frequency (0 = *low sampling* *frequency*, 1 = *high sampling frequency*) as a Level 2 predictor of the random intercepts and the random slopes of the day of the study (Model 1). The equations for Model 1 for predicting the momentary emotional clarity of person *i* at measurement occasion *t* were

- Level 1:

1 $$Clarit{y}_{ti}={\pi }_{0i}+{\pi }_{1i} day\; of\; study\;+{e}_{ti}$$

- Level 2:

2 $${\pi }_{0i}={\beta }_{00}+{\beta }_{01} \;sampling\; frequency\;+{r}_{0i}$$

3 $${\pi }_{1i}={\beta }_{10}+{\beta }_{11} \;sampling\; frequency+{r}_{1i}$$

where the fixed effect β_00_ is the expected average momentary emotional clarity on the first day of the study in the low sampling frequency group. The difference between the two sampling frequency groups in momentary emotional clarity on day 1 is represented by β_01_. β_10_ characterizes the daily change in momentary emotional clarity in the low sampling frequency group. The difference between the two sampling frequency groups in the daily change in momentary emotional clarity (cross-level interaction) is represented by β_11_ (test of Hypothesis 1).

For momentary pleasant-unpleasant mood as the dependent variable, we again began with an unconditional growth curve model (with the day of the study as the only predictor; Model 2) to analyze whether momentary pleasant-unpleasant mood changed within participants over the course of the study. To test Hypothesis 2a (moderator effect of dispositional mood regulation), we entered (grand-mean-centered) dispositional mood regulation as a Level 2 predictor of the random intercepts and the random slopes of the day of the study (Model 2a). The equations for Model 2a were

- Level 1:

4 $$Moo{d}_{ti}={\pi }_{0i}+{\pi }_{1i} \;day\; of\; study+{e}_{ti}$$

- Level 2:

5 $${\pi }_{0i}={\beta }_{00}+{\beta }_{01} \;mood\; regulation+{r}_{0i}$$

6 $${\pi }_{1i}={\beta }_{10}+{\beta }_{11}\; mood\; regulation+{r}_{1i}$$

where the fixed effect β_00_ is the expected average momentary pleasant-unpleasant mood on the first day of the study for individuals with average mood regulation. β_01_ characterizes the relationship between mood regulation and momentary pleasant-unpleasant mood on day 1. β_10_ characterizes the daily change in momentary pleasant-unpleasant mood for individuals with average mood regulation. β_11_ is the effect of mood regulation on the daily rate of change in pleasant-unpleasant mood (cross-level interaction; test of Hypothesis 2a).

To test Hypothesis 2b (whether the moderating effect of mood regulation would be stronger for the high sampling frequency group than for the low sampling frequency group), we entered the two-way interaction between mood regulation and sampling frequency as an additional Level 2 predictor of the random intercepts and the random slopes of day of study (Model 2b). The equations for Model 2b were

- Level 1:

7 $$Moo{d}_{ti}={\pi }_{0i}+{\pi }_{1i}\;day \;of\; study+{e}_{ti}$$

- Level 2:

8 $$\begin{array}{lc}{\pi }_{0i}={\beta }_{00}+{\beta }_{01}\;mood\; regulation+{\beta }_{02} sampling\; frequency\;+\\ \;\;\;\;\;\;\;\;\;{ \beta }_{03}\;mood \;regulation \times sampling\; frequency+{r}_{0i}\end{array}$$

9 $$\begin{array}{lc}{\pi }_{0i}={\beta }_{10}+{\beta }_{11}\;mood\; regulation+{\beta }_{12} sampling\; frequency\;+\\\;\;\;\;\;\;\;\;\; { \beta }_{13}\; mood\; regulation \times sampling\; frequency+{r}_{1i}\end{array}$$

where the fixed effect β_00_ is the expected average momentary pleasant-unpleasant mood on day 1 in the low sampling frequency group for individuals with an average level of mood regulation. β_01_ characterizes the relationship between mood regulation and momentary pleasant-unpleasant mood on day 1 in the low sampling frequency group. β_02_ characterizes the difference between the two sampling frequency groups in momentary pleasant-unpleasant mood on day 1 for individuals with average mood regulation. β_03_ represents the difference between the two sampling frequency groups in the relationship between mood regulation and momentary pleasant-unpleasant mood on day 1. β_10_ is the expected daily change in momentary pleasant-unpleasant mood in the low sampling frequency group for individuals with average mood regulation. β_11_ is the effect of mood regulation on the daily rate of change in pleasant-unpleasant mood in the low sampling frequency group. β_12_ is the difference between the two sampling frequency groups in the daily rate of change in pleasant-unpleasant mood for individuals with average mood regulation. β_13_ represents the difference between the two sampling frequency groups in the effect of mood regulation on the daily rate of change in pleasant-unpleasant mood (test of Hypothesis 2b).

Separate models were run for the two mood regulation dimensions (negative mood repair and positive mood maintenance; indicated by the subscripts _NMR_ and _PMM_). In addition to the preregistered analyses, we exploratively tested whether the results were similar when the actual number of completed measurement occasions (after careless responding screening) was used as a predictor of the varying slope coefficients instead of the sampling frequency group. The main analyses were computed with R, Version 4.2.2 (R Core Team, 2022). All multilevel models were created with the R package lme4, Version 1.1-30 (Bates et al., 2015), and *p* values were computed with the R package lmerTest, Version 3.1-3 (Kuznetsova et al., 2017). As an effect size, we calculated the proportion of total outcome variance explained by predictors via fixed slopes ${R}_{t}^{2(f)}$ (Rights & Sterba, 2019) with the R package r2mlm, Version 0.3.3 (Shaw et al., 2023). The within- and between-person correlations of the Level 1 variables were computed in Mplus, Version 8.9 (Muthén & Muthén, 1998–2023).

### Transparency and openness

We report how we determined our sample size, all data exclusions, all manipulations, and all measures in the study. The data and analysis code underlying this publication are publicly available at doi: 10.17605/OSF.IO/VW3GF. All hypotheses, the study’s design and its analysis were preregistered on the OSF under 10.17605/OSF.IO/JBF7W.^4^

## Results

Table 1 presents the descriptive statistics and bivariate correlations. On occasions in which individuals were in a more pleasant mood, their momentary emotional clarity was higher (within-person correlation). Moreover, at the between-person level, mean emotional clarity was positively associated with mean pleasant-unpleasant mood and both dispositional mood regulation dimensions (between-person correlations). Individuals higher in mood regulation showed more pleasant-unpleasant mood across occasions. All correlations were similar in magnitude between the experimental groups.

**Table 1:** Descriptive statistics and bivariate correlations for the main variables presented separately for each experimental group

	Low sampling frequency				High sampling frequency			
	1	2	3	4	1	2	3	4
1) Momentary emotional clarity	*-*	.23 [.16, .30]			-	.17 [.11, .24]		
2) Momentary pleasant-unpleasant mood	.54 [.41, .67]	-			.49 [.36, .62]	*-*		
3) Negative mood repair	.37 [.21, .53]	.48 [.36, .60]	-		.41 [.28, .54]	.43 [.31, .55]	-	
4) Positive mood maintenance	.32 [.18, .45]	.44 [.31, .57]	.49 [.38, .61]	-	.39 [.24, .54]	.44 [.29, .59]	.48 [.37, .59]	-
*n*	2142	2142	153	153	2209	6636	160	160
*M*	5.45	5.12	2.85	3.14	5.30	4.98	2.76	3.03
*SD*_within_	0.86	0.91	-	-	0.93	0.85	-	-
*SD*_between_	0.80	0.81	0.56	0.56	0.82	0.85	0.58	0.58

Between-person correlations (low sampling frequency: *N*_persons_ = 153; high sampling frequency: *N*_persons_ = 160) are presented below the diagonal. The within-person correlation between the two momentary measures (low sampling frequency: *N*_occasions_ = 2142; high sampling frequency: *N*_occasions_ = 2209) is presented above the diagonal. All correlations were significant at *p* < .001. For all daily measures, we extracted the mean (intercept) and standard deviation from the multilevel null model of the respective variable

### Change in momentary emotional clarity

**Fig. 1:**
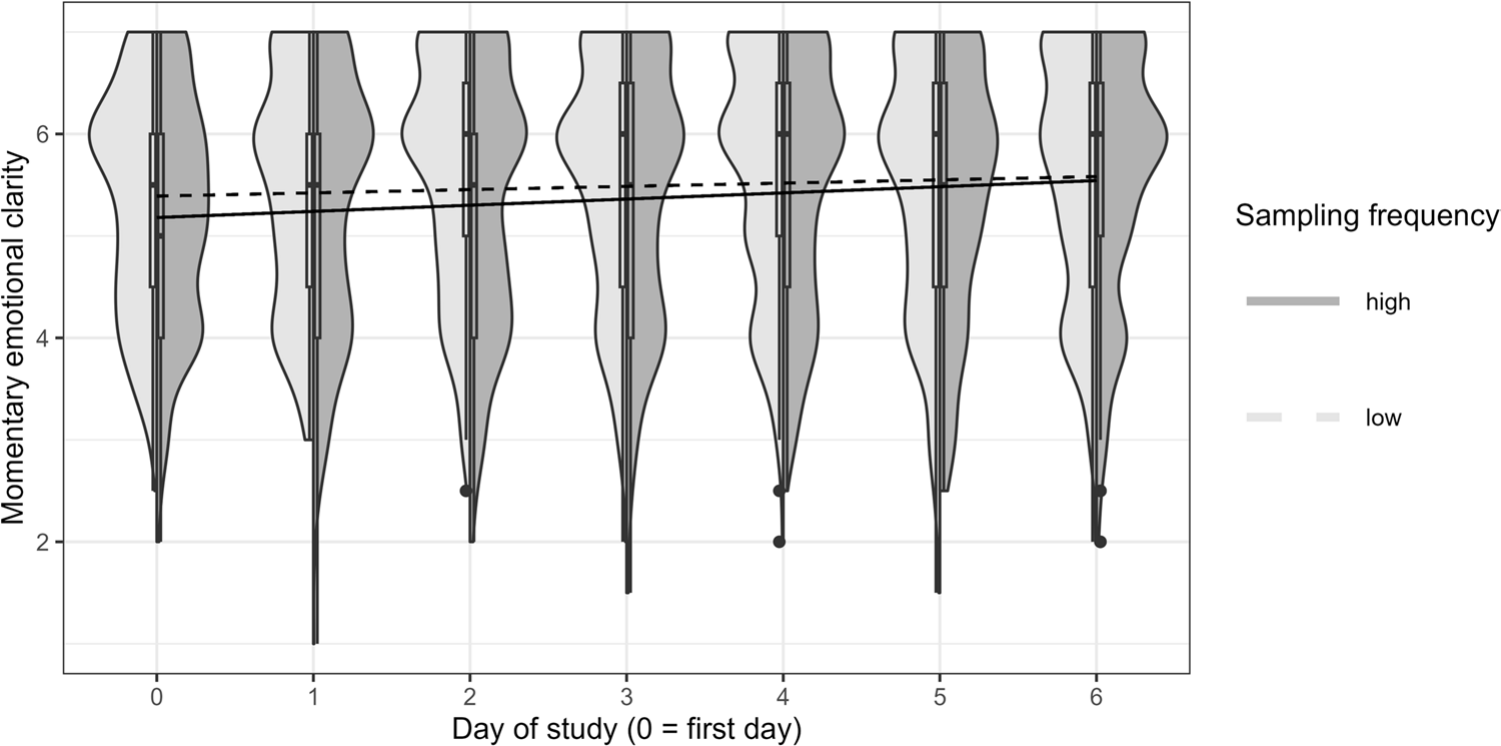
*Distribution* of momentary emotional clarity for both sampling frequency groups (*left side*: low sampling frequency, *right side*: high sampling frequency) and model-based mean intraindividual change in momentary emotional clarity over the course of 1 week (separate lines for low vs. high sampling frequency). *Note.* Individual trajectories (and observed values) of momentary emotional clarity can be found in Figure S1 in the online supplemental material

Table 2 presents the results on the change in momentary emotional clarity over time. We first tested whether momentary emotional clarity changed within persons over the course of the study (fixed effect: average intraindividual change over time). The results revealed that momentary emotional clarity increased within participants over the course of the study (Model 0). Individuals differed in this intraindividual change over time, with 65% of the participants showing an increase in momentary emotional clarity over time, and 35% of participants showing a decrease over time (Hox, 2010). The average intraindividual increase was 0.05 per day on a scale ranging from 1 to 7 (0.28 across the whole week).^5^ Contrary to Hypothesis 1, the experimental groups (low vs. high sampling frequency) did not differ in intraindividual change in momentary emotional clarity over time (nonsignificant coefficient β_11_ from Model 1, Fig. 1). Unexpectedly, the high sampling frequency group showed lower momentary emotional clarity on the first day of the study (significant coefficient β_01_ from Model 1). To rule out the possibility that the randomization had failed, we checked whether the two experimental groups differed in dispositional emotional clarity. Participants assigned to the low and high sampling frequency groups showed comparable levels of dispositional emotional clarity (*M*_low_ _sampling frequency_ = 3.08, *SD* = 0.60; *M*_high_ _sampling frequency_ = 3.03, *SD* = 0.66), *t*(311) = 0.72, *p* = .472, 95% CI [– 0.09, 0.19], *d* = 0.08.

**Table 2:** Fixed effects for multilevel models predicting momentary emotional clarity (Hypothesis 1)

Outcome Predictor	Coef.	Estimate (*SE*)	95% CI	*t*	*df*	*p*	Slopes > 0^a^
Model 0: Momentary emotional clarity							
Intercept		5.24 (0.05)	[5.13, 5.34]				
Day of study (L1)		0.05 (0.01)	[0.03, 0.07]	4.68	277.5	< .001	65%
Model 1: Momentary emotional clarity							
Intercept	β_00_	5.36 (0.07)	[5.21, 5.50]				
Day of study (L1)	β_10_	0.03 (0.01)	[0.004, 0.06]	2.26	275.1	.024	
Sampling frequency (L2)	β_01_	– 0.24 (0.10)	[– 0.44, – 0.04]	– 2.32	295.1	.021	
Day of study (L1) x Sampling frequency (L2)	β_11_	0.03 (0.02)	[– 0.01, 0.07]	1.44	276.6	.150	

*N*_occasions_ = 4351. Coef. = coefficient from multilevel Eqs. (1) to (3) in the text; L1 = Level 1 predictor; L2 = Level 2 predictor. The first day of the study was coded zero. The reference category for sampling frequency was the group with a low sampling frequency. The effect sizes ${R}_{t}^{2(f)}$ were .01 for Models 0 and 1

^a^ Based on the assumption of normally distributed slope coefficients, this value indicates the estimated percentage of slope coefficients that are positive (Hox, 2010)

Additionally, we exploratively tested whether the results were similar when the actual number of completed measurement occasions (after careless responding screening) was used as a predictor of the varying slope coefficients at the person level instead of the sampling frequency group. The results were similar (for details, see online supplemental material, Table S1). The only exception to this was that the number of measurement occasions was unrelated to the varying intercepts (i.e., emotional clarity at the first day of the study).

### Change in momentary pleasant-unpleasant mood

**Fig. 2:**
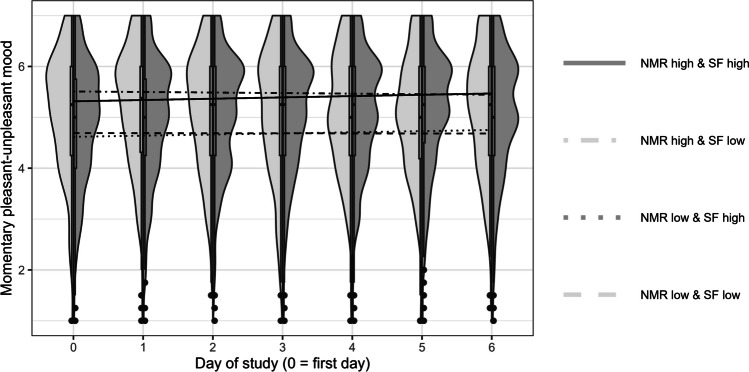
Distribution of momentary pleasant-unpleasant mood for both sampling frequency groups (*left side*: low sampling frequency, *right side*: high sampling frequency) and model-based mean intraindividual change in momentary pleasant-unpleasant mood over the course of 1 week (separate lines for low vs. high sampling frequency and low vs. high negative mood repair). *Note*. NMR = negative mood repair, SF = sampling frequency. Low negative mood repair was *M* – 1 *SD*, high negative mood repair was *M* + 1 *SD*. Individual trajectories (and observed values) of momentary pleasant-unpleasant mood can be found in Figure S2 in the online supplemental material

**Fig. 3:**
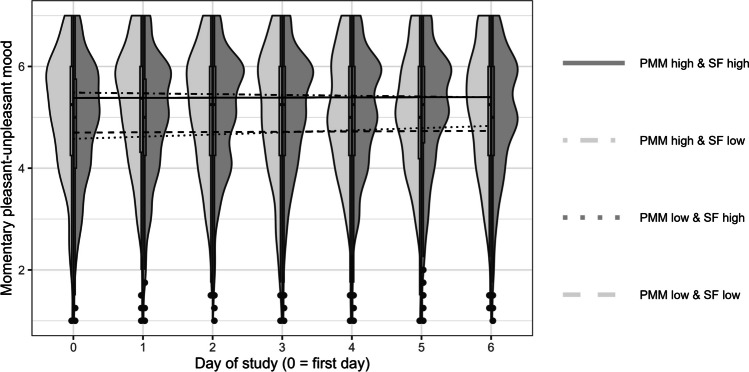
Distribution of momentary pleasant-unpleasant mood for both sampling frequency groups (*left side*: low sampling frequency, *right side*: high sampling frequency) and model-based mean intraindividual change in momentary pleasant-unpleasant mood over the course of 1 week (separate lines for low vs. high sampling frequency and low vs. high positive mood maintenance). *Note*. PMM = positive mood maintenance, SF = sampling frequency. Low positive mood maintenance was *M* – 1 *SD*, high positive mood maintenance was *M* + 1 *SD*

Table 3 presents the results on the change in momentary pleasant-unpleasant mood over time. On average, momentary pleasant-unpleasant mood did not change within participants over time (Model 2). Individuals differed in the intraindividual change over time, with 53% of the participants showing an increase in momentary pleasant-unpleasant mood over time, and 47% of participants showing a decrease in momentary pleasant-unpleasant mood over time (Hox, 2010).^6^ However, contrary to Hypothesis 2a, these individual differences in intraindividual change in mood over time could not be predicted by dispositional mood regulation, neither regarding negative mood repair (Model 2a_NMR_) nor regarding positive mood maintenance (Model 2a_PMM_). The sampling frequency groups did not differ in the daily rate of change in momentary pleasant-unpleasant mood (nonsignificant coefficient β_02_ in Models 2b_NMR_ and 2b_PMM_, Figs. 2 and 3). Contrary to Hypothesis 2b, the sampling frequency groups did not differ in the association between mood regulation and daily change in momentary pleasant-unpleasant mood (nonsignificant coefficient β_13_ in Models 2b_NMR_ and 2b_PMM_).

**Table 3:** Fixed effects for multilevel models predicting momentary pleasant-unpleasant mood (Hypothesis 2)

Outcome Predictor	Coef.	Estimate (*SE*)	95% CI	*t*	*df*	*p*	Slopes > 0^a^
Model 2: Momentary pleasant-unpleasant mood							
Intercept		5.02 (0.06)	[4.91, 5.13]				
Day of study (L1)		0.01 (0.01)	[– 0.01, 0.03]	1.21	283.1	.229	53%
Model 2a_NMR_: Momentary pleasant-unpleasant mood							
Intercept	β_00_	5.02 (0.05)	[4.91, 5.12]				
Day of study (L1)	β_10_	0.01 (0.01)	[– 0.01, 0.03]	1.10	283.2	.272	
NMR (L2)	β_01_	0.67 (0.09)	[0.49, 0.85]	7.34	300.8	<.001	
Day of study (L1) x NMR (L2)	β_11_	– 0.003 (0.02)	[– 0.04, 0.03]	– 0.19	282.8	.852	
Model 2b_NMR_: Momentary pleasant-unpleasant mood							
Intercept	β_00_	5.11 (0.08)	[4.95, 5.26]				
Day of study (L1)	β_10_	– 0.01 (0.02)	[– 0.04, 0.02]	– 0.39	376.6	.699	
NMR (L2)	β_01_	0.73 (0.14)	[0.45, 1.00]	5.18	352.6	<.001	
Sampling frequency (L2)	β_02_	– 0.16 (0.10)	[– 0.37, 0.04]	– 1.56	308.1	.119	
Day of study (L1) x NMR (L2)	β_11_	– 0.01 (0.03)	[– 0.06, 0.05]	– 0.30	371.8	.766	
Day of study (L1) x Sampling frequency (L2)	β_12_	0.03 (0.02)	[– 0.01, 0.07]	1.46	303.0	.146	
NMR (L2) x Sampling frequency (L2)	β_03_	– 0.12 (0.19)	[– 0.48, 0.24]	– 0.64	312.2	.522	
Day of study (L1) x NMR (L2) x Sampling frequency (L2)	β_13_	0.01 (0.04)	[– 0.06, 0.08]	0.31	304.5	.755	
Model 2a_PMM_: Momentary pleasant-unpleasant mood							
Intercept	β_00_	5.02 (0.05)	[4.92, 5.12]				
Day of study (L1)	β_10_	0.01 (0.01)	[– 0.01, 0.03]	1.13	282.6	.258	
PMM (L2)	β_01_	0.74 (0.09)	[0.57, 0.91]	8.35	294.8	<.001	
Day of study (L1) x PMM (L2)	β_11_	– 0.03 (0.02)	[– 0.07, 0.003]	– 1.74	276.8	.084	
Model 2b_PMM_: Momentary pleasant-unpleasant mood							
Intercept	β_00_	5.10 (0.08)	[4.95, 5.25]				
Day of study (L1)	β_10_	– 0.005 (0.02)	[– 0.04, 0.03]	– 0.32	377.8	.751	
PMM (L2)	β_01_	0.71 (0.14)	[0.44, 0.98]	5.17	349.9	<.001	
Sampling frequency (L2)	β_02_	– 0.14 (0.10)	[– 0.34, 0.06]	– 1.38	307.3	.170	
Day of study (L1) x PMM (L2)	β_11_	– 0.02 (0.03)	[– 0.07, 0.04]	– 0.67	370.3	.502	
Day of study (L1) x Sampling frequency (L2)	β_12_	0.03 (0.02)	[– 0.01, 0.07]	1.33	303.6	.185	
PMM (L2) x Sampling frequency (L2)	β_03_	0.03 (0.18)	[– 0.32, 0.38]	0.17	307.6	.867	
Day of study (L1) x PMM (L2) x Sampling frequency (L2)	β_13_	– 0.02 (0.04)	[– 0.09, 0.05]	– 0.44	301.6	.660	

*N*_occasions_ = 8778. Coef. = coefficient from multilevel Eqs. (4) to (9) in the text; L1 = Level 1 predictor; L2 = Level 2 predictor. The first day of the study was coded zero. The reference category for sampling frequency was the group with a low sampling frequency. NMR = negative mood repair; PMM = positive mood maintenance. The effect sizes ${R}_{t}^{2(f)}$ were .0004 for Model 2; .10 for Models 2a_NMR_, 2a_PMM_, and 2b_PMM_; and .09 for Model 2b_NMR_

^a^ Based on the assumption of normally distributed slope coefficients, this value indicates the estimated percentage of slope coefficients that are positive (Hox, 2010)

Again we additionally explored whether the results were similar when the actual number of completed measurement occasions (after careless responding screening) was used as a moderator instead of the sampling frequency group. The results were similar (for details, see online supplemental material, Table S2).

## Discussion

Across a 7-day AA period, we found that, on average, momentary emotional clarity increased within persons over time. By contrast, there was no mean-level change in momentary pleasant-unpleasant mood within participants over time. These findings highlight that not all constructs are equally prone to measurement reactivity. However, the two experimental groups (low vs. high sampling frequency) did not differ in the temporal course of momentary emotional clarity or momentary pleasant-unpleasant mood. On an individual level, the number of completed measurement occasions also did not moderate the temporal course of momentary emotional clarity or momentary pleasant-unpleasant mood. Finally, mood regulation did not moderate the temporal course of momentary pleasant-unpleasant mood.

Our findings that momentary emotional clarity increased within participants but momentary pleasant-unpleasant mood did not are consistent with prior findings that reactivity seems to be construct-specific. The finding that momentary emotional clarity increased is contrary to the study by Eisele et al. (2023), who found no change in momentary emotional clarity. Given the small increase in momentary emotional clarity in our study, it might be the case that the study by Eisele et al. (2023) was underpowered for such a small effect. Moreover, two studies showed an increase in emotion differentiation (Hoemann et al., 2021; Widdershoven et al., 2019), which is also a construct that is associated with the precise representation of affect. Our finding that momentary pleasant-unpleasant mood did not change over the course of our study is consistent with prior studies that found no change in positive affect (Aaron et al., 2005; Cruise et al., 1996; De Vuyst et al., 2019; Helbig et al., 2009; Husky et al., 2010) or negative affect (Aaron et al., 2005; Cruise et al., 1996; De Vuyst et al., 2019; Eisele et al., 2023; Helbig et al., 2009; Heron & Smyth, 2013; Husky et al., 2010). Barta et al. (2012) identified seven factors that might explain why reactivity occurs for some constructs but not for others: awareness and reflection, motivation, perceived desirability of the behavior, instructions or demand for change, the number of behaviors being self-monitored, sequence of monitoring, and explicit feedback. These factors make clear that whether reactivity occurs for a specific construct during a specific study depends on various details of the study design, the construct itself, and individual characteristics. Such information should be taken into account in future studies.

Regarding our experimental manipulation of sampling frequency (3 vs. 9 measurement occasions per day), we found no group differences in the temporal course of momentary emotional clarity or momentary pleasant-unpleasant mood. This finding is in line with the findings by Eisele et al. (2023), who also experimentally manipulated the sampling frequency (3 vs. 6 vs. 9 measurement occasions per day) and found no group differences in the temporal course of substantive constructs (emotional awareness, positive and negative affect, clarity, and rumination). These results suggest that the exact number of daily measurement occasions is not very decisive for potential measurement reactivity (at least in the range of 3 to 9 daily measurement occasions) and give researchers room to choose a design that is compatible with their substantive research questions.

An intraindividual increase in momentary emotional clarity over time was found for both experimental groups (low vs. high sampling frequency), which indicates that answering questions about momentary mood (and related variables) three times a day is sufficient for producing measurement reactivity. Our results are mute as to whether a reactivity effect might occur for emotional clarity at a lower sampling frequency. It would be interesting for future studies to investigate whether measurement reactivity pertaining to emotional clarity would also occur in daily diary studies with one measurement occasion per day. If this were the case, it might be helpful to include “rest days” without any assessments to circumvent measurement reactivity on emotional clarity.

In the context of research projects on momentary emotional clarity, measurement reactivity should be avoided to obtain valid data. However, our finding that momentary emotional clarity increased during the 7 AA days can be used for therapeutic practice. Emotional clarity seems to play a crucial role in emotion regulation (Lischetzke & Eid, 2017) and is associated with various mental disorders: Previous research has shown that emotional clarity was reduced in patients with somatic symptom disorders (Schnabel et al., 2022) and depression (Thompson et al., 2015). Moreover, lower emotional clarity was associated with higher depression scores (Berenbaum et al., 2012), higher posttraumatic stress symptoms (Tull et al., 2007), and various personality disorder symptoms (Leible & Snell, 2004) in subclinical or healthy populations. Hence, elements from AA studies might be combined with classical therapeutic treatment to increase levels of emotional clarity as a transdiagnostic factor (Vine & Aldao, 2014).

We derived the hypothesis that mood regulation would moderate the temporal course of pleasant-unpleasant mood on the basis of theoretical assumptions and empirical results on the effect of dispositional attention to feelings on well-being that was moderated by mood regulation (Lischetzke & Eid, 2003). This derivation rests on the assumption that asking participants multiple times per day to report on their momentary mood would generally enhance attention to feelings in daily life. Whereas momentary attention to feelings should inevitably be enhanced during AA prompts that include questions about current mood, it is unclear whether this effect also held true for the time between prompts. Future research should investigate whether repeated reporting on one’s current mood (as opposed to reporting on non-affective states or behaviors) actually has a lasting effect on attention to feelings. In addition, it might be interesting to examine in future research whether the hypothesized moderator effect of mood regulation on the temporal course of mood across an AA phase occurs only during times of stress or hardship, when individuals experience relatively frequent and intense negative emotions. Another avenue for future research might be to ensure that the studied sample includes a broader range of individual differences in dispositional mood regulation when testing for a moderator effect of dispositional mood regulation.

## Limitations

Our finding that the high sampling frequency group showed lower momentary emotional clarity on the first day of the study is surprising because participants were randomly assigned to one of the two experimental conditions. Whereas randomization might fail in small samples (Broglio, 2018; Kernan et al., 1999), our sample was large enough. Moreover, we did not observe group differences in trait emotional clarity. Hence, it is not clear why these group differences occurred.

## Constraints on generality

Our analyses were based on an all student (and predominantly female) sample. Moreover, it can be assumed that the sample was mainly Western, educated, industrialized, rich, and democratic (WEIRD, Henrich et al., 2010). Data collection took place at German universities where such sample characteristics could be expected. Whereas the internal validity regarding the analyses on the moderating role of sampling frequency should not be affected (due to randomization), it remains an open question whether the results can be generalized to other samples with, for example, a lower educational background. Previous research has shown that emotional intelligence is associated with cognitive ability (e.g., Fallon et al., 2014; Joseph & Newman, 2010). Consistent with this finding, we found relatively high levels of emotional clarity in our highly educated sample. The increase in emotional clarity over the course of an ambulatory assessment study might be even stronger in samples with lower emotional intelligence where the potential to gain more emotional clarity due to heightened self-monitoring may be higher. However, whether this assumption is true should be empirically investigated in future studies.

The present analyses were based on an AA phase of 7 days with three or nine prompts per day (depending on the sampling frequency condition). Whether our findings generalize to longer AA phases (e.g., 21 days) should be investigated in future studies. We suspect that the increase in momentary emotional clarity would be even stronger. Moreover, it might be the case that the hypothesized effects regarding the temporal course of momentary pleasant-unpleasant mood and the moderating role of mood regulation occur only in longer AA phases. The two sampling frequency groups did not differ in the temporal course of momentary emotional clarity or momentary pleasant-unpleasant mood in the present study. Sampling frequency effects on measurement reactivity might only occur when the sampling frequency conditions differ more (e.g., 2 vs. 12 prompts per day).

The present data were collected in spring 2019 and spring 2020 (during the non-lecture period between the winter semesters and the summer semesters). This was done to ensure that participants were flexible enough to reply to up to nine prompts per day. However, there might have been some exams during this period (for some participants) which might have influenced the results of the present study. Whether the results would be comparable during the lecture period should be investigated in future studies. We suppose that the number of missed prompts would increase while the substantial results should not be very different.

## Conclusion

The experimental manipulation of sampling frequency and the inclusion of mood regulation as a participant characteristic follow the recommendation to study not only mean-level changes but also the conditions under which measurement reactivity occurs in time-intensive studies (Affleck et al., 1999; Barta et al., 2012). In our study, measurement reactivity occurred for momentary emotional clarity but not for momentary pleasant-unpleasant mood. It seems desirable to further systematically investigate which psychological constructs are especially prone to measurement reactivity in AA studies, under which conditions measurement reactivity is more likely to occur (e.g., depending on the study design), and which participants might be especially susceptible to changes in the constructs being measured as a reaction to AA study participation. Similar to the recommendations offered by other researchers (e.g., Arslan et al., 2021; Eisele et al., 2023), we suggest that measurement reactivity analyses be included in future AA studies by default. Nevertheless, the statistical power also needs to be high enough (Barta et al., 2012) to find measurement reactivity effects of a certain size that would be practically relevant.

## Data Availability

The data and analysis code underlying this publication are available on the OSF (10.17605/OSF.IO/VW3GF). All hypotheses, the study’s design and its analysis were preregistered on the OSF under 10.17605/OSF.IO/JBF7W.

## Footnotes

1 To define an inconsistency index (Meade & Craig, 2012) for each measurement occasion in an AA study, items that are very similar in content and demonstrate a very large (negative or positive) within-person correlation are needed. In our study, momentary pleasant-unpleasant mood items (good-bad vs. happy-unhappy vs. unpleased-pleased vs. unwell-well; within-person intercorrelations ranged from *r* = |.64| to |.73|), momentary calm-tense mood items (tense-relaxed vs. calm-rested; the within-person correlation was *r* = – .55), and momentary wakefulness-tiredness items (tired-awake vs. rested-sleepy; the within-person correlation was *r* = – .71) met these criteria. The response format was a bipolar seven-point Likert scale with the endpoints verbally labeled (e.g., 1 = *very good* to 7 = *very bad*). Inconsistent responding at a particular measurement occasion is a response pattern that is internally/logically inconsistent. More specifically, we defined inconsistent responding as illogical responses across a mood item pair with responses near (or at) the extremes of the scale (Categories 1 or 2 vs. 6 or 7). For example, response patterns such as feeling ‘very happy’ and ‘very unwell’ at the same time or feeling ‘very happy’ and ‘very bad’ at the same time would be categorized as inconsistent responses.

2 There was one participant in the low sampling frequency group who contributed 23 measurement occasions due to a technical issue with the smartphone application.

3 To ensure that the two sampling frequency groups differed in their effective number of completed measurement occasions, we re-ran the analyses with a minimum compliance requirement of at least 21 completed occasions in the high sampling frequency group. Data from 19 participants with < 21 completed occasions were deleted. The results were very similar (online supplemental material, Tables S3 and S4).

4 Our Hypothesis 1 corresponds to Hypothesis 4 from the preregistration, and our Hypothesis 2 corresponds to Hypothesis 5 from the preregistration. Analyses regarding Hypotheses 1, 3, and 6 from the preregistration were published by Hasselhorn et al. (2022). Analyses regarding Hypothesis 2 were published by Hasselhorn et al. (2023).

5 In addition to the preregistered linear change hypothesis, we conducted exploratory tests of whether the change in momentary emotional clarity was non-linear (quadratic or exponential). There was no quadratic or exponential change in momentary emotional clarity in the low sampling frequency group (|*t*| ≤ 1.68), and there was no significant difference between the two sampling frequency groups in the quadratic or exponential coefficient (|*t*| ≤ 1.73; online supplemental material, Tables S5 and S6). To explore whether changes in momentary emotional clarity occurred on a day-to-day basis (e.g., larger change from day 1 to day 2 and smaller changes on subsequent days), we ran a discontinuous model (Singer & Willett, 2003), thus allowing day-to-day changes to differ between days. This model showed that the change rates between two consecutive days were not significantly different in the low sampling frequency group (|*t*| ≤ 1.69), and again, there were no significant differences between the two sampling frequency groups in these change rate differences (|*t*| ≤ 1.64; online supplemental material, Table S8).

6 In addition to the preregistered linear change hypothesis, we conducted exploratory tests of whether the change in momentary pleasant-unpleasant mood was non-linear (quadratic or exponential). While there was no change in the low sampling frequency group (neither linear, nor quadratic), there was a quadratic change in the high sampling frequency group (β_day.linear_ = 0.09, *t* = 2.50, *df* = 199.5, *p* = .013; β_day.quadratic_ = -0.01, *t* = -1.99, *df* = 198.1, *p* = .047; online supplemental material, Table S5), with the highest pleasant-unpleasant mood on day 5. However, compared to a linear model, the increase in ${R}_{t}^{2(f)}$ was only .001. Therefore, the linear model was retained to avoid overfitting. There was no exponential change in momentary pleasant-unpleasant in the low sampling frequency group (*t* = -0.28) and there was no significant difference between the two sampling frequency groups in the exponential change coefficient (*t* = 1.26; online supplemental material, Table S6). To explore whether changes in momentary pleasant-unpleasant mood occurred on a day-to-day basis, we fitted a discontinuous model (Singer & Willett, 2003), allowing day-to-day changes to differ between days. This model showed that the change rates between two consecutive days were not significantly different in the low sampling frequency group (|*t*| ≤ 1.70), and again, there were no significant differences between the two sampling frequency groups in these change rate differences (|*t*| ≤ 1.86; online supplemental material, Table S8).
